
JOURNAL INFORMATION
==============================
NLM Title Abbreviation: Buffalo Med J Mon Rev Med Surg Sci
NLM Journal ID: 0004276

The Buffalo Medical Journal and Monthly Review of Medical and Surgical Science

Publisher: Biomedical Journal Digitization Project

ARTICLE INFORMATION
==============================
PMCID: PMC8932453
PMID: 35376950
Article version: 1
Subjects: Editorial Department

Case of Imperforate Rectum

Print publication date: 1858 Dec
Volume: 14
Issue: 7
First page: 441
Last page: 443
License: This work is free of known copyright restrictions. For more information, please see PMC Back Issue Digitization.
License URL: https://creativecommons.org/publicdomain/mark/1.0/

==============================

which a habit of stimulating obtains upon the organism is of such a character, that it requires the strongest will to give up a habit, which an abused constitution reminds him every moment that he has made almost a physical necessity. The impossibility of legislating successfully against this evil, has been abundantly proven; we, however, can do something, as has been shown by the success in many instances of institutions for inebriates in other countries. Such a movement on the part of the profession in this country, will reflect upon them a lasting honor, and we all hope and pray it may be successful.

---

*Case of Imperforate Rectum.* Letter from Dr. Jones, of Charlotte, Monroe County, N. Y.

Editor of Buffalo Medical Journal.

Dear Sir: I have met with a very remarkable case in my practice, and if you think it worth publishing, you are at liberty to do so.

On September 18th, 1858, Mrs. Nelson was delivered, after a short labor, of a fine healthy boy, weighing nine and a-half pounds, plump and fat. The child seemed quite well for two days, nursed two or three times, slept well, and was quiet with the exception of occasional turns of vomiting a dark slimy substance, as infants frequently do at that age. After two days, we thought it should have its bowels evacuated, and therefore administered a laxative, which was speedily rejected. Another was given, but also rejected. The vomiting continued. bringing up a dark offensive substance, resembling meconium. We then commenced with enemata, which were not retained a moment. After having made repeated injections, failing to get any feces, I introduced a bougie about two and a-half inches, into the rectum, when I met with an obstruction. After ten days, the parents thought an operation might be performed to relieve the child, and Dr. Carpenter was called in. After examining the rectum, we passed a small gum-elastic catheter up the bowel, as we thought, about eight inches. Dr. C. thought it should remain for four or five hours. At the expiration of that time I removed it and found that it had coiled itself in the recium, which was very easily explained when the instrument became warm and soft. I judged from this, and previous examinations, that there was a permanent obstruction in the rectum. After twelve days suffering, the child keeping nothing on its stomach, and nothing passing its bowels, it died on the morning of the twelfth day.

I obtained the consent of the parents to make a post-mortem examination, which was done in the presence of two old ladies. The stomach and bowels were in a normal state until I came to where the sigmoid flexure of the c lon should have been, and here was wanting about three inches of intestine. The end of the colon was smoothly sealed over, making its termination in a bag. Over the upper end of the rectum was a strong thick membrane. The kidneys, ureters, bladder and urethra, were normal. The bowels were very much distended with gas. I was permitted to make no further examination.

I am pleased that I was allowed to make the examination, on account of the gossiping of a certain homœopathist, who said, "he could have cured the child, *that he had had a hundred such cases*." I cannot say that I have had eleven hundred obstetrical cases, but I have never met with such a case before. I have read of them and presume they have been met with frequently.

Yours, &c.,

A. JONES, M. D.

The above is indeed an interesting case. Imperforate rectum is quite a rare disease, and with the exception of a few cases where the bowel terminates just beneath the skin, are not amenable to an operation. We believe, indeed, that most surgeons recommend to leave them alone, and of course death is the inevitable result. In this case, we conceive that the post-mortem examination proved that an operation would have been unsuccessful. The alternative of an artificial anus is too disgusting to be taken for a moment into consideration. Conceive of the responsibility of entailing such a loathesome disease upon a fellow creature! Who is there who would not a thousand times prefer to have died in infancy? The subject of operation in cases of imperforate rectum was brought up a short time since, before the Boston Society for Medical Improvement. We have not the reports at hand, but think that in most cases the operations were unsuccessful.

In another point of view, the case reported by Dr. Jones, gives us a text. The meddlesome interference of irregular practitioners, who are always too ready to play upon the hopes of the friends of patients laboring under a serious disease. This precious homœopathist could cure the child, "he had cured a hundred such cases." This is the invariable cry, and often are the minds of the friends filled with remorse, as well as the standing of the practitioner injured, by such bare-faced effrontery. A post-mortem examination, however, will bring the truth to light, and it not only defends the reputation of the practitioner, but must be a great relief to friends who otherwise might not have believed that all had been done.

It is only a few days ago that a patient from whom we had removed the matrix of the nail of the great toe, for onychia maligna, after the nail had previously been removed by the ordinary operation, and after she had suffered for three year-, told us that a homœopathic practitioner volunteered the valuable information, that now her toe was healed up she would die of consumption. This *professional gentleman* was working with her husband in a blacksmith's shop, only a short time before, and from thence proceeded to practice medicine, when he had promised to cure her toe himself with the inevitable sugar of milk. To be classed with such animals is humiliating, but it is one of the crosses which we knew we should have to bear when we took up our profession, and we suppose we have no right to complain.

---

*Meeting of the Medical Society of South-Western New York.*—Every half year we receive an account of a meeting of this society, and judge from what we see that it is always a pleasant affair. When doctors feel a disposition to come together as brethren, they are the most companionable of men; good feeling alone prevails, and this appears always to be the case with our brethren of South-Western New York.

The society met on the first Wednesday in November, and elected the following gentlemen officers for the ensuing year:

*President*, H. M. T. Smith, Dunkirk.
*Vice-President*, Edson Boyd, Ashville.
*Secretary and Treasurer*, Charles K. Irwin, Dunkirk.
*Executive Committee*, Dr. G. C. Bennet, Quincy,
" A. K. Avery, Forestville,
" G. S. Harrison, Sinclearville.
*Com. of Arrangements for* 1859, Dr. Boyd, Ashville,
Dr. Thomas, Dewittville,
" Fenwick, Mayville.

The following resolutions, among others, were unanimously adopted:

*Resolved*, That the members of the Medical Society of South-Western New York will not, in any instance, visit, examine or prescribe for a patient of an irregular practitioner of medicine, until the patient, or his or her guard-
